# COVID-19 Exposure, Treatment Patterns, and Healthcare Utilization in US Long-Term Care: A Retrospective EHR Study

**DOI:** 10.1093/geroni/igaf122.3907

**Published:** 2025-12-31

**Authors:** Sohini Ganguli, Brittin Wagner, Florin Draica, Daniel Verdi, Kate Mathers, Ralph Valeriote, Stefan Gravenstein

**Affiliations:** Shionogi Inc, Florham Park, New Jersey, United States; PointClickCare, Bloomington, Minnesota, United States; Shionogi Inc, Florham Park, New Jersey, United States; Shionogi Inc, Florham Park, New Jersey, United States; PointClickCare, Mississauga, Ontario, Canada; PointClickCare, Mississauga, Ontario, Canada; Alpert Medical School of Brown University, Providence, Rhode Island, United States

## Abstract

Long-term care (LTC) residents remain disproportionately affected by COVID-19 due to age-related vulnerabilities and complex comorbidities. Here we characterize COVID-19 exposure, treatment, and healthcare resource utilization (HCRU) in U.S. LTC facilities using a large-scale electronic health record (EHR) database. We conducted a retrospective observational study using PointClickCare’s Life Sciences EHR database (2022–2025). COVID-19 exposure, diagnosis, and treatment events were identified using ICD-10 codes and medication orders. Outcomes included demographic and clinical characteristics, antiviral treatment patterns, contraindicated medication use, and HCRU metrics. Among 1.4 million COVID-19-related events documented in the EHR, we found 143,943 exposures, 932,821 diagnoses, and 350,421 treatments. Residents with documented exposures to COVID-19 were younger (mean age 72) than those diagnosed (77) or treated (79). Just under 20% of medication orders for COVID-19 antiviral treatment were associated with EHR documented diagnosis within 14 days. Paxlovid was the most prescribed antiviral, though ∼43% of treatments occurred without an EHR-documented diagnosis or exposure. Medication adjustments to reduce interactions between chronic disease medications and antiviral treatments were made for approximately one-third of patients on a contraindicated medication (35% on Paxlovid and 28% on molnupiravir). COVID diagnosis was associated with increased MACE, hospitalizations, and oxygen use, especially in the first week following diagnosis. This study highlights critical gaps in exposure documentation and antiviral treatment uptake in LTC settings. Findings underscore the need for improved coding practices, prescriber education, and understanding timely post-exposure prophylaxis strategies to reduce clinical burden and improve outcomes for LTC residents.

